# The Application of Pulmonary Ultrasound in Neonatal Ventilator-Associated Pneumonia

**DOI:** 10.3389/fped.2022.882056

**Published:** 2022-06-29

**Authors:** Peng Jiang, Jing Wei

**Affiliations:** ^1^Department of Paediatrics, Liaocheng People’s Hospital, Liaocheng, China; ^2^Department of Ultrasonography, Liaocheng People’s Hospital, Liaocheng, China

**Keywords:** infant, newborn, ultrasonic examination, lung, ventilator-associated pneumonia

## Abstract

This study explored the application value of lung ultrasound (LUS) in neonatal ventilator-associated pneumonia (NVAP). In this study, 122 newborns suspected of NVAP were treated in the NICU of Liaocheng People’s Hospital between July 1, 2020, and July 1, 2021. Of these, 115 were clinically diagnosed with NVAP. The diagnostic value of LUS for NVAP was determined by comparing the different signs of LUS and chest X-ray (CXR). The confirmed cases were divided into the failure and success groups according to the first ventilator weaning test results. The consistency between the results of LUS and CXR and the actual test results was compared between the two groups. Before treatment, the LUS findings of the confirmed cases showed a lung consolidation with air bronchogram sign (111/115), alveolar-interstitial syndrome (113/115), pleural effusion (12/115), pleural line abnormalities (114/115), and lung pulse (15/115). CXR showed 109 cases of pneumonia. Taking the clinical diagnosis of VAP as the gold standard, the lung consolidation with air bronchogram sign on LUS had a higher sensitivity, specificity, and accuracy for the diagnosis of NVAP than those of other LUS and CXR findings and showed better consistency with the clinical diagnosis (*AUC* = 0.983, kappa value = 0.761, *p* < 0.05). After treatment, the 115 cases were divided into two groups according to the results of the first weaning from ventilation: the failed group (19 cases) and the successful group (96 cases). The lung consolidation with air bronchogram sign was used as the positive diagnostic standard of ultrasound. The sensitivity and specificity of LUS (94.7 and 89.6%, respectively) in evaluating the outcome of weaning from the ventilator of pneumonia were higher than those of CXR (73.7 and 84.4%, respectively). Additionally, the consistency of the LUS findings with the weaning results was higher than that of CXR (*AUC* = 0.922, kappa value = 0.709, *p* < 0.05). Therefore, compared with CXR, LUS has a higher value in diagnosing NVAP and can better predict the results of the ventilator off-line test. LUS can replace CXR as the first imaging examination for NVAP.

## Introduction

Ventilator-associated pneumonia (VAP) refers to pneumonia that occurs in patients who have undergone endotracheal intubation or tracheotomy within 48 h after mechanical ventilation and 48 h after weaning and extubation (1). Being a major form of nosocomial infection and cause of death (2), prolonged mechanical ventilation, and increased medical expenses, neonatal VAP (NVAP) is a severe complication of mechanical ventilation in neonates, and its incidence is greater than 30% in China (2). Pediatricians diagnose NVAP in combination with patients’ clinical manifestations, imaging, and laboratory examination. At present, the imaging examination of NVAP is mainly based on the chest X-ray (CXR) examination results. Fabregas et al. showed that the sensitivity and specificity of CXR for clinical diagnosis of VAP were only 69 and 75%, respectively (3). Bedside CXR examination is fast and convenient, and it was routinely used for the examination of pneumonia in the past. However, the radiation (4), difficulty in disinfecting, poor image quality, and low resolution make it easy to miss diagnoses (5), which limits its diagnostic value for NVAP. Computed tomography can evaluate the pulmonary morphology and ventilation treatment effect of NVAP patients, and it has a high diagnostic value. However, the clinical application of CT is limited due to the high transport risk of critically ill infants and the high radiation exposure dose. lung ultrasound (LUS) technology, having been developed rapidly in recent years, provides a new tool for the diagnosis and monitoring of neonatal pulmonary inflammation, and it has the advantages of convenience, ease of disinfection, no radiation (6), and ease of repetition.

Authoritative experts have reported that a lung consolidation with an air bronchogram sign is the main ultrasonic sign of neonatal infectious pneumonia (7). Given that the lung condition of critically ill children receiving mechanical ventilation is more complex and several factors can lead to changes in LUS imaging findings, the values of different LUS signs in the diagnosis and efficacy evaluation of NVAP are worth discussing. This study explored the values of different LUS signs to provide the basis for the diagnosis of NVAP and the selection of ventilator withdrawal time.

## Materials and Methods

### Patients

This study selected 122 patients suspected of NVAP who received mechanical ventilation in the neonatal intensive care unit of Liaocheng People’s Hospital between July 1, 2020, and July 1, 2021. The inclusion criteria were as follows: (1) having relevant respiratory symptoms or signs; (2) being hospitalized for more than 48 h and having received mechanical ventilation for no less than 48 h; and (3) being less than 28 days old. The exclusion criteria were: (1) severe congenital heart diseases, such as tetralogy of Fallot and complete transposition of great arteries; (2) disseminated intravascular coagulation; (3) multiple organ failure; (4) blood system diseases; and (5) serious surgical disease. This study was approved by the ethics committee of Liaocheng People’s Hospital, and the legal guardians of the infants signed the informed consent form.

### Neonatal Ventilator-Associated Pneumonia Diagnostic Criteria

There is no recognized gold standard for the diagnosis of NVAP. Every patient with suspected NVAP can be diagnosed only after combining their medical history, physical examination, and corresponding auxiliary examination.

According to the clinical diagnostic criteria (8), NVAP is diagnosed if one of the following conditions is met: (1) mechanical ventilation treatment is performed for non-pulmonary infectious diseases and the pathogen culture of airway secretion is positive 48 h after treatment, or new pathogens are cultured 48 h after original pulmonary infection treatment with mechanical ventilation; (2) although the pathogen culture of airway secretions was negative, the following symptoms and signs of pulmonary infection occurred clinically: increased body temperature, significant changes in airway secretions, increased wet rales in the lungs, increased infection of peripheral blood leukocytes, and infiltration shadow on lung X-ray films; (3) although there was no new pathogen in the culture of airway secretion, there were signs of secondary pulmonary infection in the clinic.

### Judgment Criteria for the Results of Ventilator Withdrawal Test

In our study, clinicians determined whether to withdraw the ventilation machine based on various observations and auxiliary examinations. Weaning was successful if the patient could tolerate spontaneous breathing within 48 h after weaning and did not need mechanical ventilation to facilitate breathing. If the patient had obvious respiratory distress, unstable circulation, cyanosis, and poor response within 6 h after weaning from ventilation (9) or could not maintain spontaneous breathing within 72 h after the test and still needed mechanical ventilation, the test was considered to have failed (10, 11).

### Instruments and Methods

The LUS and X-ray examination were performed within 12 h after the newborn was suspected of NVAP and before the ventilator weaning test, and the examination results were recorded. LUS and CXR inspection should be conducted within 1 h. The two examinations were performed and interpreted by two senior imaging doctors with professional training. The positive X-ray findings were new or progressive patchy infiltrating shadows in the lungs.

The LUS was performed according to the international guidelines for neonatal LUS (2019) (12). We used the MylabTwice ultrasound instrument (Esaote, Italy) and a high-frequency linear array probe (frequency 10–12 Mhz). The infants were positioned in the supine, lateral, or prone positions in a quiet state. Each side of the lung of the newborn was divided into six areas: anterior upper, anterior lower, lateral upper, lateral lower, posterior upper, and posterior lower. During the examination, the probe was perpendicular to the chest wall, and 12 areas of both lungs were scanned longitudinally along each intercostal space. Static and dynamic images were saved, and each area was marked. Personnel and instrument probes were disinfected and isolated during the examination. The examiner was unaware of the newborn’s condition.

**FIGURE 1:**
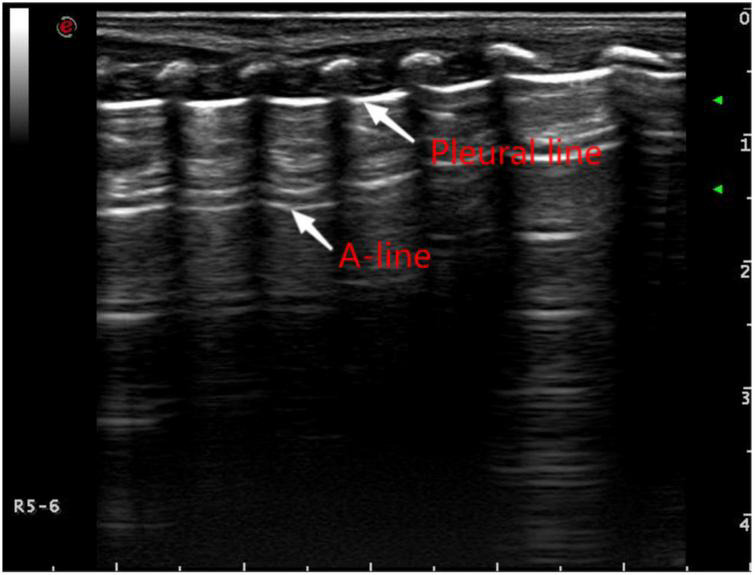
Pulmonary ultrasound findings of normal newborns. Pulmonary ultrasound shows that the pleural line and A-line are clear, smooth, and parallel like “bamboo knots.”

**FIGURE 2:**
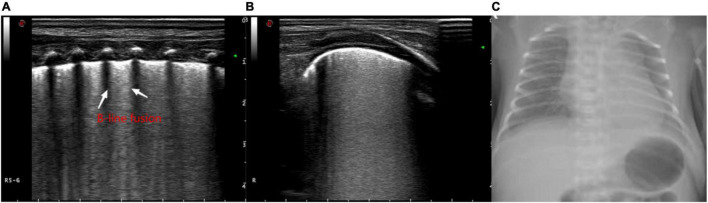
The ultrasonographic manifestations in the lungs of a newborn with alveolar-interstitial syndrome, showing the increase of B lines and the fusion into “white lung” like changes. **(A)** Longitudinal image of the intercostals. **(B)** Transverse image along the intercostals. **(C)** X-ray manifestation of the decrease of light transmission in both lungs.

**FIGURE 3:**
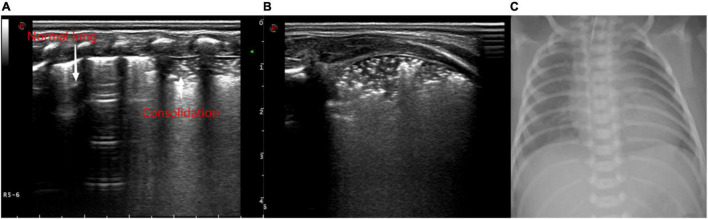
Ultrasonic manifestations of neonatal lung consolidation. Pulmonary ultrasound shows an irregular consolidation area with a hyperechoic air bronchogram sign. It shows that dot or linear hyperechoic artifacts vary with breathing in the lung consolidation tissue and pleural lines. Normal lung tissue can be observed at its location. **(A)** Longitudinal image of intercostals. **(B)** Transverse image of intercostals. **(C)** X-ray showing multiple patchy fuzzy shadows in both lungs.

**FIGURE 4:**
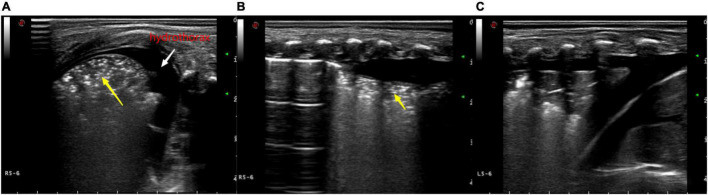
Ultrasonic manifestations of neonatal pulmonary consolidation combined with pleural effusion. Pulmonary ultrasound shows consolidation area with air bronchogram (yellow arrow). A-line disappears, and pleural effusion can be observed. **(A)** Longitudinal image of the intercostals. **(B,C)** Transverse image along the intercostals.

**FIGURE 5:**
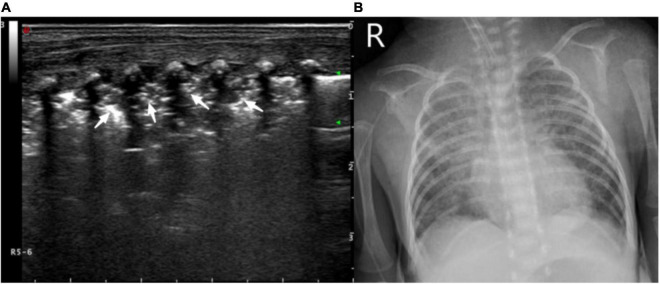
**(A)** Ultrasound manifestations of large-area consolidation of newborn lungs. Pulmonary ultrasound shows a large irregular consolidation area with air bronchogram (white arrow) and the disappearance of the pleural line and A-line. The disappearance of the pulmonary slip and lung pulse can be observed with real-time ultrasound. **(B)** X-ray showing a diffuse patchy fuzzy shadow of both lungs and a bronchogram of lower field.

**FIGURE 6:**
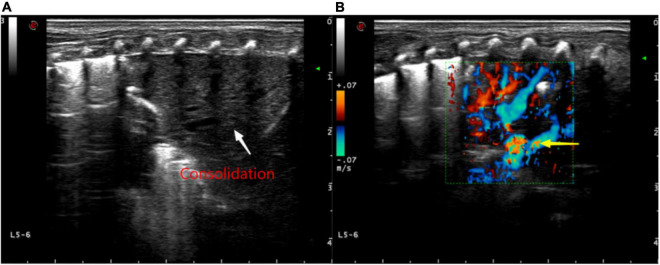
**(A)** Ultrasound findings of a large consolidation in areas 5–6 of the neonatal left lung. Lung ultrasound shows a large area with the “tissue-like sign” similar to the echo of the liver, spleen, and other tissues (white arrow) and the disappearance of the pleural line and A-line. **(B)** Color Doppler ultrasound showing blood flow with “dendritic” distribution in the area of consolidation (yellow arrow).

Ultrasound examination was mainly used to observe the ultrasound findings, such as A line (Figure 1), B line, alveolar-interstitial syndrome (Figure 2), lung consolidation with air bronchogram sign (Figure 3), pleural effusion (Figure 4), pleural line abnormalities (Figure 5), pulmonary pulsation syndrome (Figure 5), and the internal blood supply of lung consolidation (Figure 6) (10).

### Statistical Analysis

We used SPSS 20.0 to sort out and analyze the data. The values of LUS and CXR for the diagnosis of NVAP were determined based on sensitivity, specificity, and accuracy. The kappa test was used to evaluate the CXR and LUS results with the clinical diagnosis and weaning results. Kappa test and receiver operating characteristic curve (ROC curve) were used to evaluate the consistencies and diagnostic efficacies of CXR and LUS results with clinical diagnosis and weaning results. We adopted the χ2 test, and *p* < 0.05 denoted statistical significance.

## Results

### General Clinical Information

Among 122 infants suspected of NVAP, 115 were clinically diagnosed with NVAP, including 60 men and 55 women whose gestational ages were 27–40 + 2 weeks and onset dates were 3–7 days after birth. There were 69 premature infants and 46 full-term infants; cesarean section was performed for 59 of them, and 56 were born through vaginal delivery. Their birth weights ranged from 1050 to 4000 g.

Among the 115 cases of NVAP diagnosed clinically, LUS showed the lung consolidation with air bronchogram sign for 111 cases, alveolar-interstitial syndrome for 113 cases, pleural effusion for 12 cases, pleural line abnormalities for 114 cases, and pulmonary pulsation syndrome for 15 cases; CXR showed pneumonia for 109 cases. Seven newborns with NVAP were not clinically diagnosed; 4 had neonatal transient tachypnea syndrome (TTN), 2 had apnea, and 1 had pleural effusion. On LUS examination, 5 had alveolar-interstitial syndrome, 1 had pleural effusion, and 1 had an abnormal pleural line; 2 newborns had a positive CXR.

### Comparison of Different Pulmonary Ultrasonic Signs and Chest X-Ray Findings for the Diagnosis of Neonatal Ventilator-Associated Pneumonia

**FIGURE 7:**
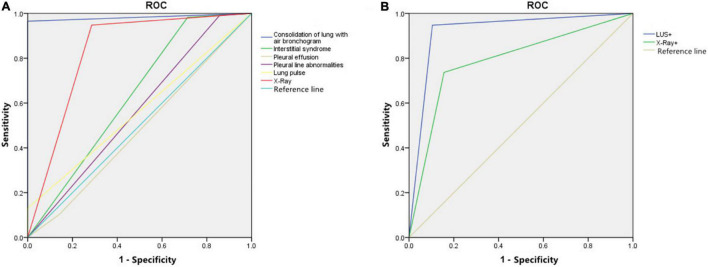
**(A)** The receiver operating characteristic (ROC) curve of different lung ultrasound (LUS) signs and chest X-ray (CXR) in the diagnosis of neonatal ventilator-associated pneumonia (NVAP). **(B)** The ROC curve of LUS and CXR findings for the prediction of NVAP weaning outcome.

Using the clinical diagnosis of NVAP as the gold standard, the sensitivity, specificity, accuracy, kappa value, and the area under the ROC curve (AUC) of each LUS sign and CXR diagnosis of NVAP are shown in Table 1. The ROC curve is shown in Figure 7.

**TABLE 1:** Comparison of different pulmonary ultrasound signs and chest X-ray (CXR) findings for the diagnosis of neonatal ventilator-associated pneumonia (NVAP).

Examination method		Clinical diagnosis of NVAP (cases)		Sensitivity (%)	Specificity (%)	Accuracy (%)	AUC	Kappa value	*P*-value
		Positive (*n* = 115)	Negative (*n* = 7)						
Ultrasound sign	Lung consolidation with air bronchogram	111	0	96.5	100	96.7	0.983	0.761	*P* < 0.05
	Interstitial syndrome	113	5	98.3	28.6	94.3	0.634	0.336	*P* < 0.05
	Pleural effusion	12	1	10.4	85.7	14.8	0.481	−0.005	*P* > 0.05
	Pleural line abnormalities	114	6	99.1	14.3	94.3	0.567	0.202	*P* < 0.05
	Lung pulse	15	0	13.0	100	18.0	0.565	0.017	*P* > 0.05
X-ray findings		109	2	94.8	71.4	93.4	0.831	0.522	*P* < 0.05

The sensitivity, specificity, and accuracy of lung consolidation with the bronchial inflation sign for the diagnosis of NVAP based on LUS were 96.5, 100.0, and 96.7%, respectively. The kappa value (0.761, *p* < 0.05) and the AUC (0.983) of this LUS sign were higher than that for other LUS signs and the CXR finding (kappa value = 0.522, *p* < 0.05); therefore, when the positive signs of LUS and CXR were used alone in the diagnosis of VAP, the diagnostic value of lung consolidation with bronchial inflation sign in LUS was the highest. The LUS findings were more consistent with the clinical diagnosis of NVAP.

The specificity (100%) of the LUS finding of pulmonary pulsation for the diagnosis of NVAP was significantly higher than that for CXR (71.4%) but the sensitivity and accuracy were low. The sensitivity and accuracy of the LUS findings of alveolar-interstitial syndrome (98.3 and 94.3%, respectively) with pleural line abnormalities (99.1 and 94.3%, respectively) for the diagnosis of NVAP were slightly higher than those of CXR (94.8 and 93.4%) but the specificity (28.6 and 14.3%) was significantly lower than that of CXR (71.4%). The kappa values of interstitial syndrome, pleural line abnormalities, and lung pulse were greater than 0 but lower than for the CXR findings, and the diagnostic value of LUS for NVAP was lower than that of CXR. The AUC of these pulmonary ultrasonic signs was less than 0.7 with low accuracy in the diagnosis of NVAP.

### Comparison of Consistency Between Pulmonary Ultrasound and Chest X-Ray Findings and Actual Results of Ventilation Weaning

After clinical treatment, 115 NVAP cases were divided into the failure and success groups according to the results of the first ventilator withdrawal test: failure group, 19 cases; success group, 96 cases.

The lung consolidation with bronchial inflation sign, which showed the highest consistency with the clinical diagnosis, was selected as the positive diagnostic standard for LUS. In the failure group, 18 cases were positive and 1 case was negative for the LUS exam, while 14 cases of CXR were positive and 5 cases were negative. In the success group, 10 and 86 cases were positive and negative based on LUS, while 15 and 81 cases showed positive and negative CXR results, respectively.

The sensitivity and specificity of LUS (94.7 and 89.6%, respectively) in evaluating the outcome of the ventilator weaning test in patients with NVAP were higher than those of CXR (73.7 and 84.2%, respectively). The ROC curve of LUS and CXR findings for the prediction of test results is shown in Figure 7. When LUS and CXR findings were used alone to predict the test results of NVAP, the AUC and kappa value of LUS were higher than those of CXR (*p* < 0.05). The kappa value of LUS was higher than that of CXR, which was consistent with the actual test results (*p* < 0.05; Table 2). Compared with CXR, LUS can better predict the results of ventilator weaning test.

**TABLE 2:** Comparison of the consistencies of the pulmonary ultrasound and chest X-ray (CXR) findings and the actual results of ventilation weaning.

Group	Examination results (cases)		Sensitivity (%)	Specificity (%)	AUC	Kappa value	*P*-value
	Positive	Negative					
Failure group	18	1	94.7	89.6	0.922	0.709	*P* < 0.05
Success group	10	86					
Failure group	14	5	73.7	84.4	0.790	0.479	*P* < 0.05
Success group	15	81					

## Discussion

Neonatal ventilator-associated pneumonia is a severe complication of mechanical ventilation, especially in newborns with low resistance. It restricts the effect of mechanical ventilation, increases the risk of bronchopulmonary dysplasia in premature infants, significantly prolongs the hospitalization time, and increases the hospitalization cost. Invasive mechanical ventilation can damage the airway barrier of newborn children, while positive pressure ventilation causes the spread of pathogenic bacteria in the upper respiratory tract in the trachea and bronchus. During the early stage, there are inflammatory lesions centered on the pulmonary peripheral bronchioles, and these can spread to all pulmonary lobes, especially the lower lobe. Bronchopneumonia can spread outward to the peripheral alveoli and pleura, which can result in different lung gas-liquid ratios (11) and reduced pulmonary ventilation function (13), and abnormal B-line can be observed during LUS examination. With the progress of pulmonary inflammation, inflammatory lesions fuse, and ultrasound shows lung consolidation under the pleura or involving a large lung lobe (14). When the gas in the bronchus or bronchioles in the solid lung tissue has not been fully absorbed, ultrasound will produce a strong echo shadow when encountering the gas, which is manifested as a scattered or linear strong echo shadow in the solid lung tissue, which is the bronchial inflation sign. The bronchial inflation sign may be categorized as static or dynamic depending on whether the strong echo changes with respiratory movement.

Pulmonary ultrasound is widely used for the diagnosis and treatment of neonatal lung diseases (15), especially those with severe neonatal pulmonary diseases (7, 16, 17). Our study used the clinical diagnosis as the gold standard. The sensitivity, specificity, and accuracy of LUS combined with air bronchogram in the diagnosis of NVAP are higher than those of CXR, which is consistent with reports in the literature (7). Due to the thin chest wall, small lung volume, and thin subcutaneous fat layer and muscle layer of newborns, LUS is better than X-ray in scanning the lung surface and pleural effusion. Another reason may be that newborns lie on their backs for a long time, which causes pathogens to accumulate in the back under the action of gravity; therefore, abnormal signs are more common in the back. Ultrasound can be used to examine the lung through the back, and X-rays can only be taken in the supine position, while image overlaps may lead to missed diagnoses of back lesions (5).

Our study showed that the LUS findings of lung consolidation with air bronchogram and lung pulse were specific signs for the diagnosis of VAP. Lung pulse is closely related to the severity of lung consolidation and heartbeat intensity. It is mainly observed in severe pneumonia or atelectasis, and the incidence is low for NVAP. Among the 115 confirmed cases, the incidence of pulmonary pulsation (13.0%) was lower than that of lung consolidation with air bronchogram (96.5%). Therefore, the lung consolidation with air bronchogram sign had a higher clinical diagnostic value than pulmonary pulsation. Alveolar-interstitial syndrome and pleural abnormalities have high sensitivity and accuracy in the diagnosis of NVAP, but low specificity, which can also be observed for other lung diseases such as TTN. Among the 7 children with NVAP that were not clinically diagnosed, 5 had alveolar-interstitial syndrome, 1 had pleural effusion, and 1 had an abnormal pleural line. After clinical diagnosis, 4 had TTN, 2 had apnea, and 1 had pleural effusion. The main clinical manifestation of children with TTN is dyspnea, which is pathologically associated with pulmonary edema and often complicated with pleural effusion. LUS often shows alveolar-interstitial syndrome, an abnormal pleural line, and double lung points, among others (18), but TTN and pneumothorax can be distinguished by lung consolidation and bronchial inflation syndrome on LUS.

Approximately one-third to half of the infants receiving mechanical ventilation will have ventilator dependence. If weaning from ventilation fails, the risk of death can increase (19). Delayed weaning from ventilation can also increase the risks of neonatal neurodevelopmental disorders and delayed sepsis (20). Some scholars have used the LUS scoring method to evaluate the weaning time (18). However, some authoritative experts have shared concerns that ultrasound scoring cannot accurately reflect the severity of lung diseases and predict the weaning time of mechanical ventilation in newborns. Therefore, the ultrasound scoring system is not recommended for ultrasound diagnosis and curative effect evaluation of neonatal lung diseases (21). In this study, we selected the lung consolidation with air bronchogram sign as the positive diagnostic standard of LUS to evaluate the relationship between the positive rate of LUS after weaning and the results of weaning from ventilation. The positive rate of LUS in the failed group was as high as 94.7%, and that in the successful group was only 10.4%. The kappa value of LUS was higher than that of CXR. The results of the LUS examination had better consistency with the actual results of weaning from ventilation; therefore, LUS assisted the clinical prediction of the weaning outcomes. Pulmonary ultrasound findings, especially lung consolidation with the bronchial inflation sign, suggest persistent severe inflammatory exudation in the lungs of newborns, and this affects the pulmonary ventilation function, which is related to the failure of the ventilator removal test. Clinicians can use it to dynamically evaluate the condition and changes of pulmonary inflammation in newborns, choose better weaning times, and adjust and optimize clinical treatment.

Compared with lung X-ray, LUS is more sensitive, and LUS can detect a small area of atelectasis in almost any part of the lung, regardless of the location (22). Pediatricians can help newborns choose the appropriate body position according to the lesion location and judge whether patients need to have their backs patted and sputum extracted. However, LUS also has limitations. They are as follows: the near-field attenuation of pulmonary ultrasound can affect the far-field diagnosis; a large number of gas reflection and bone shielding may cause inexperienced ultrasound doctors to fail to observe the focus, which requires doctors to constantly improve their scanning methods and comprehensively observe the lungs by changing the position and angle of the ultrasonic probe; for the clinical diagnosis of ventilator-associated pneumonia with ultrasound, a complementary diagnosis based on laboratory inflammatory indexes is needed.

The study also has limitations. We did not analyze the outcome and prognosis of the children. In addition, the number of samples selected in this study is small, and there are only 19 cases in the weaning failure group. In the future, we can conduct prospective, multicenter, and large-scale clinical research to confirm the accuracy of the research results. In addition, we will further study the feasibility of using LUS-assisted clinics to guide NVAP patients to wean off ventilators. We hope that LUS can further assist pediatricians in treating critically ill children.

## Conclusion

In conclusion, as an auxiliary examination, pulmonary ultrasound is simple, reproducible, and radiation-free. It can be used for real-time dynamic evaluation of pulmonary morphological changes in NVAP and has several advantages over other detection methods. LUS can facilitate the dynamic evaluation of NVAP and help guide clinical weaning. We are confident that LUS will become the first-line imaging examination for the diagnosis of NVAP in the future.

## Data Availability Statement

The raw data supporting the conclusions of this article will be made available by the authors, without undue reservation.

## Ethics Statement

The studies involving human participants were reviewed and approved by the Ethics Committee of Liaocheng People’s Hospital. Written informed consent to participate in this study was provided by the participants’ legal guardian/next of kin.

## Author Contributions

All authors listed have made a substantial, direct, and intellectual contribution to the work and approved it for publication.

## Conflict of Interest

The authors declare that the research was conducted in the absence of any commercial or financial relationships that could be construed as a potential conflict of interest.

## Publisher’s Note

All claims expressed in this article are solely those of the authors and do not necessarily represent those of their affiliated organizations, or those of the publisher, the editors and the reviewers. Any product that may be evaluated in this article, or claim that may be made by its manufacturer, is not guaranteed or endorsed by the publisher.
